# Membrane Assembly and Ion Transport Ability of a Fluorinated Nanopore

**DOI:** 10.1371/journal.pone.0166587

**Published:** 2016-11-11

**Authors:** Raphaël Godbout, Sébastien Légaré, Maud Auger, Claudia Carpentier, François Otis, Michèle Auger, Patrick Lagüe, Normand Voyer

**Affiliations:** 1 Département de chimie, Regroupement québécois de recherche sur la fonction, la structure et l'ingénierie des protéines (PROTEO), Université Laval, Québec, Québec, Canada; 2 Département de biochimie, microbiologie et bio-informatique, Regroupement québécois de recherche sur la fonction, la structure et l'ingénierie des protéines (PROTEO), Université Laval, Québec, Québec, Canada; 3 Département de chimie, Regroupement québécois de recherche sur la fonction, la structure et l'ingénierie des protéines (PROTEO), Centre de recherche sur les matériaux avancés (CERMA), Centre québécois sur les matériaux fonctionnels (CQMF), Université Laval, Québec, Québec, Canada; Universidad de Santiago de Compostela, SPAIN

## Abstract

A novel 21-residue peptide incorporating six fluorinated amino acids was prepared. It was designed to fold into an amphiphilic alpha helical structure of nanoscale length with one hydrophobic face and one fluorinated face. The formation of a fluorous interface serves as the main vector for the formation of a superstructure in a bilayer membrane. Fluorescence assays showed this ion channel's ability to facilitate the translocation of alkali metal ions through a phospholipid membrane, with selectivity for sodium ions. Computational studies showed that a tetramer structure is the most probable and stable supramolecular assembly for the active ion channel structure. The results illustrate the possibility of exploiting multiple Fδ^-^:M^+^ interactions for ion transport and using fluorous interfaces to create functional nanostructures.

## Introduction

Natural ion channels have a wide variety of vital functions, including neuronal information transmission, cell communication and muscle contraction [1,2]. Ion channels operate by facilitating and regulating the flow of sodium, potassium or chloride ions in and out of cells. Developing artificial nanostructures that can mimic natural ion channel transport processes is of great interest [3].

Synthetic nanopores that could facilitate selective ion diffusion across different membranes could find applications in areas ranging from medicine to energy storage and environmental chemistry [3,4]. Although functional artificial ion channels have been prepared successfully using very elegant approaches [5–12], there is still a need to design robust nanopores, to find new ways to control their synthesis, and to optimize how they facilitate ion translocation across lipophilic membranes.

Fluorinated amino acids are increasingly used in bioactive peptides and proteins to modulate and improve their properties [13]. Due to its strong NMR signal, the fluorine atom can also be used as a probe to study biochemical processes [14]. Fluorous non-covalent interactions have attracted considerable attention recently in protein design [15–17] and in supramolecular chemistry [18–20]. An interesting property of fluorinated compounds is their tendency to segregate themselves, often leading to an entirely separate fluorous phase [21,22].

Herein, we report the use of fluorous interactions to drive the self-assembly of a highly fluorinated α-helical peptide into a well-defined superstructure in a bilayer membrane. We also demonstrate that the superstructure thus formed facilitates the translocation of alkali metal ions to act as an artificial ion channel.

## Results and Discussion

### Design

**Fig 1:**
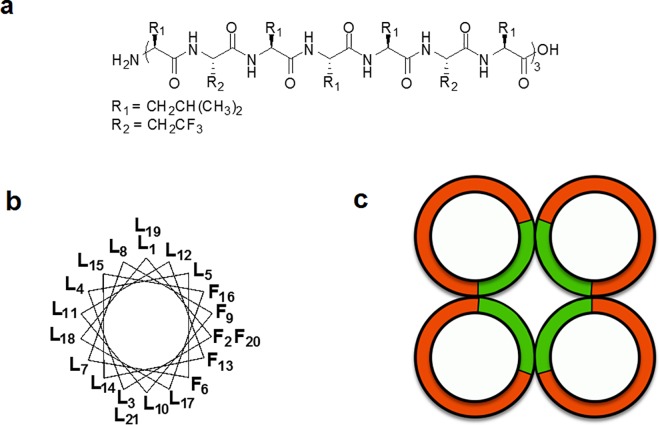
**Structure representations** (a) Linear structure of the fluorinated 21-residue peptide LX2 under investigation, with 15 L-leucines (L) and 6 L-2, 2, 2-trifluoroethylglycine (F); (b) Axial wheel projection illustrating the amphiphilic fluorous/hydrophobic faces of LX2 α-helix; (c) Schematic representation of a tetrameric arrangement of LX2, showing in green the fluorinated faces of the four-helix bundle, and in red the hydrophobic contours.

Fluorinated aminoacids have been used to stabilize artifical proteins, most interestingly four α-helical superstructures [23–27]. Inspired by those reports and based on the pioneering work of DeGrado [28] and Montal [29,30], we have designed and synthesized a fluorinated 21-residue leucine-based peptide, "*LX2*," which incorporates six (S)-2-amino-4,4,4-trifluorobutyric acid molecules. It has trifluoromethyl groups on its side-chain at positions 2, 6, 9, 13, 16 and 20, in an *I*, *I* +4, *I* +3 relationship (Fig 1A). This peptide with serine instead of the fluorinated amino acid was shown to adopt a strong α-helical conformation in membranes and to self-assemble into an efficient ion channel superstructure [31]. In a helical conformation, peptide LX2 forms an amphiphilic structure of 3.2 nm, long enough to span the hydrophobic portion of a typical bilayer membrane, with one face of the helix being hydrophobic and the other face being fluorinated (Fig 1B). When incorporated into low-polarity phospholipid bilayer membranes, we hypothesized that fluorine-fluorine interactions would favor the formation of self-assembled superstructures. This is illustrated schematically in Fig 1C.

### Peptide preparation and characterization

**Fig 2:**
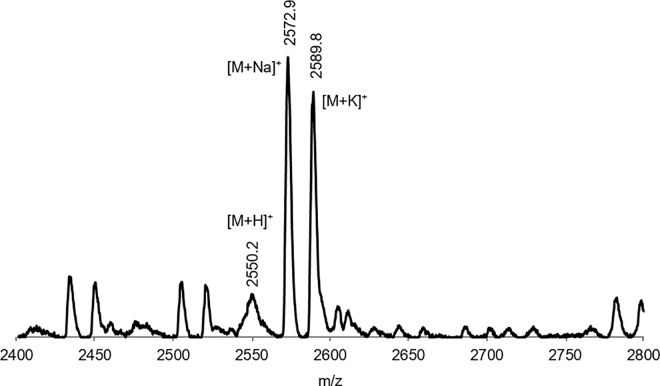
Mass spectrum of LX2 obtained using MALDI mass spectrometry.

Conveniently, LX2 can be prepared using well-established solid-phase peptide synthesis procedures. The fluorinated peptide was prepared efficiently using the Wang resin with fluorenylmethyloxycarbonyl (Fmoc)-protected amino acids. Established conditions [32] were used in the coupling of leucine and the fluorinated amino acid. For the fluorinated amino acid couplings, the more effective N-HATU [33] was used instead of N-HBTU as coupling reagent. Also, couplings were performed for two hours instead of one for leucine. Cleavage of the 21-residue peptide from the resin was achieved using 95% TFA. MALDI mass spectrometry analyses confirm the structure of LX2, with molecular peaks at 2550.2 [LX2 + H]^+^, 2572.9 [LX2 + Na]^+^ and 2589.8 [LX2 + K]^+^ (Fig 2).

Notably, LX2 could not be chromatographed using a variety of conditions and several reverse-phase columns. It appears that its propensity for self-assembling is too strong, forbidding its study by HPLC. Although the exact purity of LX2 could not be established with certainty, it is clear from MALDI mass spectrometry studies that it is over 90%.

### Conformational studies

**Fig 3:**
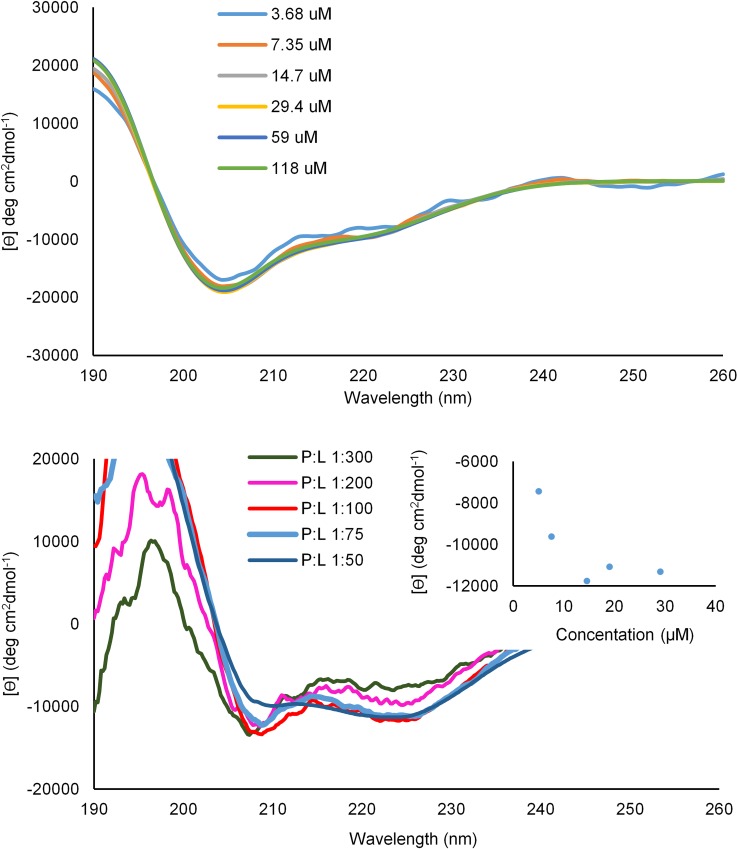
Circular dichroism spectra of LX2 Top: in HFIP at different concentrations; Bottom: using POPC/cholesterol (8:2) vesicles at different peptide/lipid ratios; Insert: Graph of molar ellipticity at 222 nm vs concentration.

We studied the conformational behavior of LX2 using circular dichroism spectropolarimetry (CD). Hexafluoroisopropanol (HFIP) was used first since the fluorinated peptide is not soluble in most of the usual solvents acceptable for CD studies. As proposed in the initial design, the most preferred conformation of LX2 is the α-helical conformation, evidenced by the typical minima at 208 nm and 222 nm (Fig 3, top). This conformational analysis is further supported by FTIR investigations in HFIP, which demonstrate the absence of β-sheet structures (see Supporting Information). Variable concentration studies in HFIP demonstrated that ellipticity at 222 nm is independent of the concentration. This suggests that LX2 does not assemble in that solvent.

Performing CD experiments using POPC/cholesterol vesicles instead showed that the helical content increased non-linearly with the concentration (Fig 3, bottom), which strongly suggests that LX2 forms self-assembled superstructures in lipid bilayer membranes. Solubility and sensitivity limited the range of concentrations that could be studied, and data obtained could not be fitted to a monomer-multimer equilibrium (Fig 3, insert). The helical content in bilayer membranes was estimated to be near 50% using CONTINLL method in DichroWeb online analysis [34,35].

### Ion transport studies

Encouraged by the self-assembling tendency of LX2 and its adoption of a strong helical conformation, we verified that it could self-assemble in lipid bilayer membranes to create nanopores able to facilitate the transport of ions. We used fluorescence kinetic assays, where the rate of fluorescence increase is measured over time [36]. Different alkali salts and concentrations of LX2 were studied. 8-hydroxypyrene-1,3,6-trisulfonic acid trisodium salt (HPTS) was chosen as pH-dependent fluorescent probe [19,32], solubilized in a 7.2 pH buffer and encapsulated in egg yolk phosphatidylcholine (EYPC) vesicles along with 100 mM of a specific salt. After addition of a base to raise the extravesicular pH to ~8.2 to create an ionic gradient, the translocation of ions could be monitored by an increase of fluorescence.

**Fig 4:**
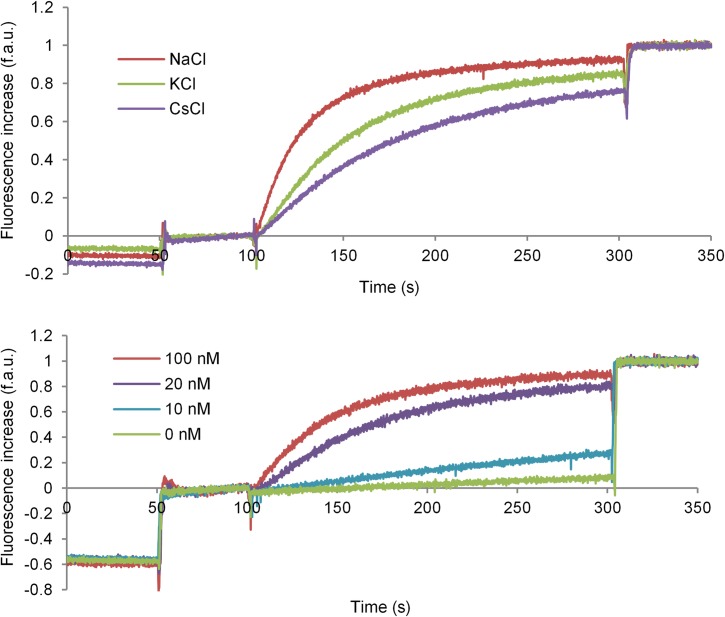
Results of ion transport assays with LX2 Top: for various chloride salts at a constant concentration of 100 nM of LX2; Bottom: with NaCl at various concentrations of LX2. Intensity of fluorescence is reported in fluorescence arbitrary units (f.a.u.).

In a typical assay, LX2 peptide was added to the vesicle solution at 100 s and the fluorescence measured. The 100% of fluorescence was defined by the maximum fluorescence observed at 350 s, after addition of Triton X-100 to lyse all vesicles to release their contents. Results of ion transport experiments are shown in Fig 4 and the rates of transport are given in Table 1.

**Table 1:** Initial rates of transport for alkali metal chlorides by LX2 at 100 nM.

	Initial rate (10^−2^ f.a.u./s)	Relative initial rate
**NaCl**	2.3 ± 0.3	1.0 ± 0.1
**KCl**	1.3 ± 0.3	0.6 ± 0.1
**CsCl**	0.8 ± 0.2	0.4 ± 0.1

Reported in fluorescence arbitrary units per second (f.a.u./s) with standard deviations.

For sodium ion transport assays, a minimum of at least 10 nM of LX2 was required to observe any significant ion translocation, indicating that a threshold concentration is required to induce the formation of a functional self-assembled superstructure (Fig 4, bottom). The rate of transport increases non-linearly with concentration for the transport of sodium chloride, as expected for a self-assembled active structure. Using concentrations over 100 nM leads only to a slight increase in the rate of transport. Transport studies also show that sodium ions are transported across the membrane more efficiently than potassium or cesium ions, as shown in Table 1. Using cations larger than sodium leads to lower transport rates, as demonstrated by the significant decreases in rates for potassium and cesium chloride (2.27 to 1.27 to 0.84 f.a.u./s. respectively using 100 nM of LX2). Additional transport assays were performed with sodium bromide and iodide to investigate the importance of the counter anions on the transport through the channel formed by LX2 (Table 2) in a membrane. At the concentrations tested, the rates of transport for NaCl and NaBr are similar within experimental error, from 2.27 to 2.46 f.a.u./s., and lower in the case of NaI (0.74 f.a.u./s.). Overall, the ion transport results confirm that Na^+^ ions are preferred over K^+^ and Cs^+^. They also demonstrate that the choice of counter anion affects the rates of transport.

**Table 2:** Transport rates for different sodium salts by LX2 at 100 nM.

	Initial rate (10^−2^ f.a.u./s)	Relative initial rate
**NaCl**	2.3 ± 0.3	1.0 ± 0.1
**NaBr**	2.5 ± 0.1	1.08 ± 0.04
**NaI**	0.74 ± 0.04	0.33 ± 0.02

Reported in fluorescence arbitrary units per second (f.a.u./s) with standard deviations.

However, the similar transport rates of NaCl and NaBr support the transport of Na^+^. The ion selectivity observed is probably due to size restriction rather than the energy of desolvation [37,38]. Indeed, it is easier to remove water surrounding larger cations, in some cases by more than 80 kJ mol^-1^ in favor of potassium against sodium for the first sphere of solvation, their respective effective radii goes from 138 to 102 pm [39]. While it should be easier for potassium to be desolvated, its passage through the water-depleted pore is disfavored as compared to Na^+^. Alternatively, the different rates in potassium, sodium or cesium transport could also be due to LX2 solubility or to its ability to diffuse into the membrane. On the other hand, the mechanism by which the iodide counter anions affect the ion translocation through the bilayer is difficult to explain. More experiments are required to elucidate the molecular details of this phenomenon.

### Lysis assays

Calcein leakage experiments [40] confirmed that the increase in fluorescence observed in the ion transport assays truly originates from the nanopore created by the assembly of LX2 units and not from simple membrane perturbation. Our results are reported in the supplemental information section. Even at a concentration of LX2 thirty times higher than the most concentrated one used in transport assays, no increase of fluorescence was noted. These results demonstrate that LX2 has no lytic ability and confirms its ion transport ability.

### *In silico* studies

**Fig 5:**
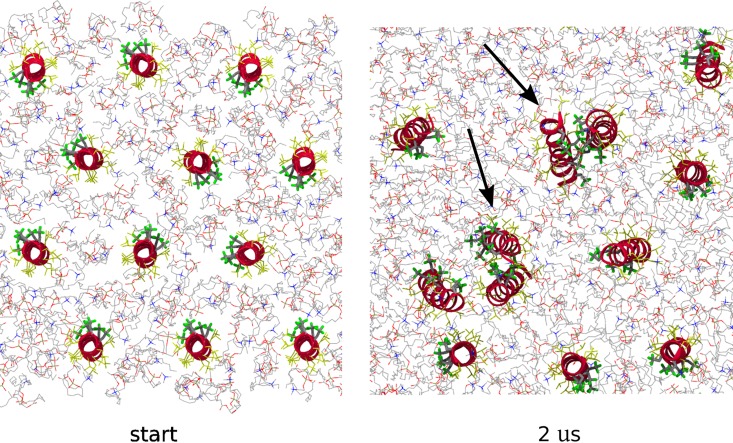
Self-assembly of LX2 dimers during MD simulations of 12 monomers. Fluorine atoms in green, carbon atoms from fluorinated amino acids in grey, peptide backbone in red and leucine residues in yellow. Lipids are shown in thin lines. Dimers are indicated by arrows.

We used *in silico* modelling techniques to study the active supramolecular assembly. First, we simulated twelve LX2 transmembrane monomers in a POPC bilayer for 2 μs. These simulations demonstrated that LX2, in a low-polarity environment, assembles into dimeric structures formed through fluorous-fluorous interactions (Fig 5). These results suggest that the formation of a fluorous interphase is highly favorable even in a low- polarity environment such as a lipid bilayer membrane.

**Fig 6:**
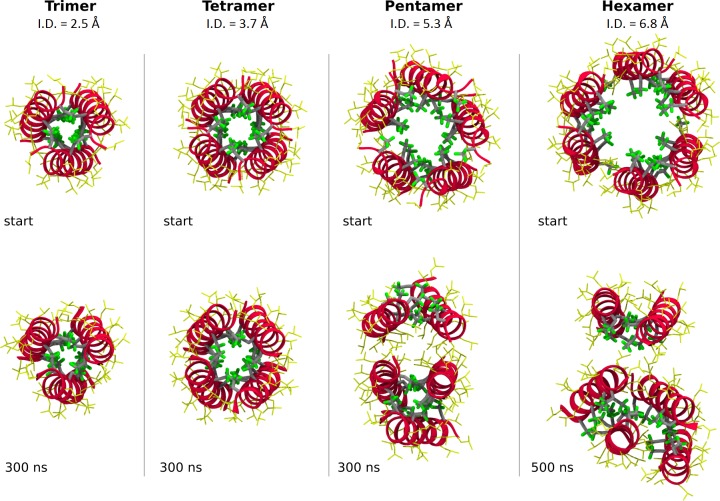
Superstructure stability (top row) and typical equilibrated structures (bottom row) of the LX2 trimer, tetramer, pentamer and hexamer. Surrounding lipids are not shown for clarity. The pentamer and hexamer starting structures are not completely symmetric following energy minimization. Internal diameters (I.D.) are averaged values along the complete pore lumen. The color code is the same as in Fig 5.

Second, the simulations were started with pre-formed superstructures of three to six LX2 units (Fig 6, top). The tri- and tetramer exhibited a high stability over time, retaining their starting superstructures almost completely intact for the whole duration of the simulations (Fig 6, bottom). By contrast, starting the simulations with a pentamer or hexamer led the units to disassemble rapidly into dimer and trimer subunits without reverting to a larger superstructure (Fig 6, bottom).

In agreement with the idea of the LX2 functional superstructure stabilization by fluorous interphases, the average fluorine-fluorine surface of contact per peptide unit was higher for the stable tetramer and lower for the stable trimer and unstable pentamer and hexamer (Table 3).

**Table 3:** Averages of the fluorine-fluorine surface of contact in Å^2^.

	Overall superstructure	Per peptide unit
**Trimer**	784.4 ± 1.2	261.5 ± 0.4
**Tetramer**	1121.9 ± 2.1	280.5 ± 0.5
**Pentamer**	1088.2 ± 4.1	217.6 ± 0.8

Errors represent standard errors.

Noteworthy of mention, the interiors of LX2 trimer and tetramer superstructures are completely devoid of a water column. The space provided in the channel formed by the assembly of a tetramer, the largest and most stable superstructure in simulations, does not seem to, according to our studies, provide a wide enough lumen for the passage of ions (Fig 6, bottom). This is in direct contradiction with ion transport assay results, which clearly demonstrate otherwise. It is possible that dynamic fluctuations of the tetrameric superstructure permit the formation of transient pores large enough to allow the passage of Na^+^ ions.

Overall, the *in silico* studies demonstrate unambiguously that the fluorine-fluorine interactions play a major role in driving the self-assembly of LX2 superstructures and that their stability relies on the fluorous interphases between peptide units. They also support the formation of a tetrameric superstructure as the functional ion channel in bilayer membranes [41].

## Conclusion

We have shown that it is possible to exploit the unique properties of fluorine atoms to drive peptide self-assembly of a highly fluorinated peptide into a well-defined superstructure in bilayer membranes. Studies by CD confirm the strong propensity of LX2 towards α-helical conformation in solution in low-polarity environments, thus generating an amphiphilic fluorous/hydrophobic peptide unit. We also demonstrated the superstructure thus formed acts as artificial ion channel, where fluorine atoms line the inner pore and play the leading role in creating polar relay sites for cations during their membrane translocation. Fluorescence assays proved that this transport is concentration-dependent, requiring a minimum concentration of 10 nM of LX2, and that sodium ions are transported most efficiently by the ion channel formed by the assembly of LX2 units. *In silico* studies confirm that a tetramer structure is the most stable of all multimeric assemblies investigated. While di-, tri-, and tetramer formations are stable, pentamer and hexamer superstructures break down to smaller structures. Finally, *in silico* results also support the importance of fluorine-fluorine interactions, and the notion that the F-F surface of contact is the major stabilizing force for self-assembled artificial ion channel.

## Experimental Procedures

### General

Egg yolk L-α-phosphatidylcholine (EYPC) and 1-palmitoyl-2-oleoylphosphatidylcholine (POPC) were purchased from Avanti Polar Lipids (Alabaster, AL, USA) and used without further purification. Wang resin, coupling reagents and Fmoc-protected amino acids were purchased from Matrix Innovation (Québec City, QC, Canada). The resin used had a substitution level of 0.73 mmol/g benzyl alcohol groups. Fmoc-S-2-amino-4,4,4-trifluorobutyric acid was purchased from PolyPeptide Group (San Diego, CA, USA). Hexafluoroisopropanol was purchased from Mellinckrodt Baker (Phillipsburg, NJ, USA). Cesium chloride was purchased from Amresco (Solon, OH, USA). Sodium chloride was purchased from Caledon Laboratories (Georgetown, ON, Canada). Potassium chloride was purchased from BDH (Toronto, ON, Canada). 1N sodium hydroxide solution was purchased from Fisher Scientific (Fair Lawn, NJ, USA). All others chemicals were purchased from Sigma-Aldrich (Milwaukee, WI, USA). All solvents were Reagent, Spectro or HPLC grade quality, purchased commercially and used without any further purification except for dimethylformamide (degassed with N_2_) and dichloromethane (distilled over CaH_2_). Water used throughout the studies was distilled and deionized using a Barnstead NANOpurII system (Boston, MA, USA) with four purification columns. Solid-phase synthesis was performed manually using solid-phase reaction vessels equipped with coarse glass frit (ChemGlass, Vineland, NJ). Sonication was done using the Avanti Polar Lipids Liposomicator. Vesicle preparation was done using a Mini-Extruder with parts purchased from Avanti Polar Lipids, Hamilton Company (Reno, Nevada, USA) and Whatman (Little Chalfont, Buckinghamshire, UK). Mass spectra were obtained using an Agilent 6210 LC Time of Flight mass spectrometer. CD measurements were carried out on a Jasco J-715 photospectrometer. Fluorescence experiments were performed on a Varian Cary Eclipse spectrofluorometer.

### Peptide synthesis

Typical procedure for peptide synthesis on solid-phase: 3 equiv. of Fmoc-L-leucine (0.0530 g, 0.150 mmol) were activated with 3 equiv. of 1-[Bis(dimethylamino)methylene]-1H-benzotriazolium-3-oxide hexafluorophosphate (N-HBTU) (0.0569 g, 0.150 mmol) and 3 equiv. of hydroxybenzotriazole (HOBt) (0.0203 g, 0.150 mmol) in DMF, and then added to the Wang resin (0.1 g, 0.050 mmol) swollen in DMF, followed by addition of 3 equiv. of DIEA (0.026 mL, 0.150 mmol). The mixture was shaken mechanically for 1 h at room temperature. The resin was filtered and washed thoroughly with DMF (3 times), MeOH (3 times), DMF (3 times), MeOH (3 times) and then dried under vacuum. The completion of the coupling reactions was monitored by the ninhydrin test. For a coupling of Fmoc-S-2-amino-4,4,4-trifluorobutyric acid, referred as X (0.0569 g, 0.150 mmol), the activation step was done with 1-[bis(dimethylamino)methylene]-1*H*-1,2,3-triazolo[4,5-b]pyridinium-3-oxid hexafluorophosphate (N-HATU) (0.0570 g, 0.150 mmol) and HOBt (0.0203 g, 0.150 mmol) in DMF, and then added to the Wang resin swollen in DMF, followed by the addition of 3 equiv. of DIEA (0.026 mL, 0.150 mmol). The mixture was shaken mechanically for 1 h at room temperature, except in the case of the fluorinated analog, which was shaken for 2 h. The rest of the procedure is the same as a leucine coupling. If necessary, a second coupling was performed under the same conditions. The Fmoc group was deprotected by two consecutive 10 min treatments with 20% piperidine/DMF. Peptide cleavage was performed using a 95% TFA/water solution for 1 h. Filtration and lyophilisation yielded 0.0542 g (43%) of the crude peptide carboxylate characterized by mass spectrometry.

### Circular dichroism spectropolarimetry

Analyses of the LX2 in solution were performed with a solution of 0.6 mg of peptide in 2.0 mL of hexafluoroisopropanol (HFIP). This solution was diluted several times with HFIP to obtain the desired concentrations. For analyses of LX2 in vesicles, variable amounts of peptide solution in HFIP were added to 53.2 μL of POPC (5 mg/mL solution in chloroform) and 67.7 μL of cholesterol (0.5 mg/mL solution in chloroform). The solvent was evaporated and the mixture was dried under vacuum. Vesicles were hydrated with 300 μL of phosphate buffer and were sonicated. Spectra were recorded using a 0.1 cm path length quartz cell. Ten scans were collected from 190 to 260 nm with a data pitch of 0.2 nm and a scanning speed of 100 nm/min.

### Preparation of EYPC vesicles for transport assays

1 mL of a solution of egg yolk phosphatidylcholine (EYPC) in chloroform at a concentration of 25 mg/mL was dried on a rotary evaporator. The residue was further dried under vacuum for 1 h. 1 mL of a solution (nanopure water containing 1 mM of 8-hydroxypyrene-1,3,6-trisulfonic acid trisodium salt (HPTS), 100 mM of sodium chloride and 10 mM of 4-(2-hydroxyethyl)-1-piperazineethanesulfonic acid (HEPES), buffered at pH 7.2 with 1N aqueous sodium hydroxide) was added to the reaction vessel. The solution was shaken on a rotary evaporator for 30 minutes at the same angle it was dried. The resulting light-green milky solution underwent 5 freeze-thaw cycles in liquid nitrogen and was then extruded through a 0.2 μm membrane 15 times. Afterwards, the vesicle solution was purified by chromatography on Sephadex G-25 to remove the excess of HPTS and 6 mL of the first product shown by a UV lamp was collected. The eluent used contained 100 mM of sodium chloride and 10 mM of HEPES in nanopure water at pH 7.2. This final vesicle solution was used as-is.

### Typical ion transport experiment

At *t* = 0 s in a disposable plastic cuvette of 1 cm length was placed 1.95 mL of a pH 7.2 solution containing 100 mM of MX where M is sodium, potassium or cesium and X is chloride, bromide or iodide, 10 mM of HEPES and 25 μL of the vesicle solution. At *t* = 50 s, 20 μL of 0.5 N aqueous sodium hydroxide (bringing the external pH at ~8.2) was added. At *t* = 100 s, 40 μL of a 5 μM LX2 peptide solution in 3% DMSO/H_2_O was added. At *t* = 300 s, 20 μL of a solution of 10% Triton-X100 in water was added to disrupt the vesicles and get a maximum of fluorescence signal. Each salt was tested twice with data collected every 0.1 s for 350 s, at an excitation wavelength of 450 nm and an emission wavelength of 510 nm with a slit of 5 nm at 25°C. The baseline was determined under the same conditions, with the addition of a solution containing only DMSO 3% in water without LX2, at 100 s.

### Calculation of initial rates of transport (V0)

The software R [42] was used to evaluate the initial rates of transport of ions by LX2. Starting from at least 101 seconds, a set of data from each fluorescence curve was used to get a linear regression fitting the plot. The slope, in arbitrary units of fluorescence per second, was selected as the initial rate (*V*_*0*_). A dataset, different for each salt and each concentration tested, and large enough to get a reasonable coefficient of determination (*R*^2^ > 0.97), was selected and the value was optimized point by point until the maximum rate was found. The selection and the optimization were done manually. The number of data points used varied from 100 (10 seconds) for the steepest slopes, to a maximum of 300 (30 seconds) for slopes where an *R*^2^ higher than 0.97 could not be reached, and ranged from a minimum of 100.7 seconds to a maximum of 199.9 seconds. The same method was used to get the background transport (under comparative conditions without LX2) and those values were subtracted from the *V*_*0*_ previously determined to get the final and correct values reported.

### Preparation of EYPC vesicles for calcein leakage assays

1 mL of a solution of EYPC in chloroform at a concentration of 25 mg/mL was dried on a rotary evaporator. The residue was further dried in vacuum for 1 h. 1 mL of a solution (nanopure water containing 80 mM of bis-[N,N−bis(carboxymethyl)amino-methyl]fluorescein (Calcein), 5 mM of ethylenediaminetetraacetic acid (EDTA) and 100 mM of HEPES buffered at pH 7 with 1N aqueous sodium hydroxide) was added to the reaction vessel. The mixture was sonicated 1 h, then purified by chromatography with Sephadex G-25 to remove the excess of calcein and the first product as shown by a UV lamp was collected. The eluent used for this contained 200 mM of sodium chloride, 100 mM of HEPES and 5 mM of EDTA in nanopure water. The concentration in vesicle was determined with a standard Bartlett test. This last solution was used as-is.

### Typical vesicle lysis experiment

At *t* = 0 s, 900 μL of a pH 7 solution containing 200 mM of NaCl, 100 mM of HEPES, 5 mM of EDTA, and 100 μL of the vesicle solution (the final concentration of vesicles was 36 mM) was placed in a disposable 1 cm long plastic cuvette. At *t* = 50 s, 40 μL of a peptide solution was added. This solution contained LX2 starting from the least concentrated to 30 times the highest tested concentration in fluorescence assays (3000 nM). At *t* = 300 s, 20 μL of a solution of 10% Triton-X100 in water was added to disrupt the vesicles and get the maximum fluorescence signal. Each concentration was tested twice with data collected every 0.1 s for 350 s, at an excitation wavelength of 490 nm and an emission wavelength of 513 nm with a slit of 5 nm at 25°C.

### *In silico* studies

#### System generation

Three all-atom systems were built, each containing twelve LX2 monomers inserted in a POPC membrane. Each LX2 monomer was generated in an α-helical conformation, as was observed from the CD spectra, with the default N- and C-termini. The peptides were positioned with a transmembrane orientation, parallel to each other, and separated by a centre-to-centre distance of 25 Å. For each system, the peptides were randomly rotated about the membrane's normal axis in order to obtain independent starting configurations. For the three systems, membrane insertion was carried using CHARMM-GUI [43] and the bilayer contained 160 lipids. The neutralized systems had a 20 Å thick layer of water each side of the bilayer and a salt concentration of 0.1 M NaCl. Each of the final system contained about 50,000 atoms.

Three all-atom systems were built for each of the studied LX2 multimers: trimer, tetramer, pentamer and hexamer. The initial structure of each multimer was assembled manually as follows. For a given structure, the peptides were placed parallel to each other and positioned to form a symmetric multimer with trifuoromethylated alanines facing the centre of the structure. Then, the structure was refined by adjusting the centre-to-centre distance of neighbor peptides to 12 Å and the peptide helix tilts to 13°. An energy minimization was performed followed by a short gas phase MD simulation at 25 K. Each LX2 multimer was given a different rotation about the membrane normal axis in order to obtain three independent starting conformations. Each multimer was then inserted in a membrane containing 160 POPC using CHARMM-GUI [43]. Each neutralized system had a 20 Å thick layer of water on each side of the bilayer and a salt concentration of 0.1 M NaCl. Each final system contained about 45,000 atoms.

#### Molecular dynamics

The all-atom simulations were run using NAMD 2.9 software [44], a CHARMM36 force field [45,46], and a TIP3P water model [47]. The topology and force field parameters of the trifluoromethylated alanine residue are provided as supplemental information. The simulations were run for 2 μs for the systems containing twelve LX2 monomers, and for 300 ns for the LX2 tri-, tetra- and pentamer. For the hexamer, two simulations were run for 500 ns and one was extended to 950 ns. A timestep of 2 fs was used for all simulations. Constant temperature and pressure conditions were simulated using Langevin dynamics with a damping coefficient of 1/ps, a temperature of 300 K, and a Nosé-Hoover Langevin piston pressure of 1 bar. Nonbonded interactions were truncated at 12 Å with a smooth switching of the forces over the range of 10–12 Å. Long-range electrostatics were calculated via the particle mesh Ewald (PME) method [48], using a sixth-order or fourth-order interpolation and a grid spacing of ≈ 1 Å. Fourth-order interpolation was used for the simulations of twelve LX2 peptides and of LX2 hexamers. Covalent bonds involving hydrogens were kept rigid with the SETTLE algorithm for water and the SHAKE algorithm for the rest of the system.

## Supporting Information

S1 Fig FTIR of LX2 in HFIP. (TIF)

S2 Fig Ion transport for sodium salts at a constant concentration of 100 nM of LX2. (TIF)

S3 Fig Calcein release at various concentrations of LX2. (TIF)

S1 File Parametrization of the trifluoromethylated alanine. (PDF)

S2 File Topology and parameters of the trifluoromethylated alanine. (PDF)
