# Supplementary figures and images for: Membrane Assembly and Ion Transport Ability of a Fluorinated Nanopore
Source: PLoS One. 2016 Nov 11;11(11):e0166587. doi: 10.1371/journal.pone.0166587 (PMC5106009; doi:10.1371/journal.pone.0166587)

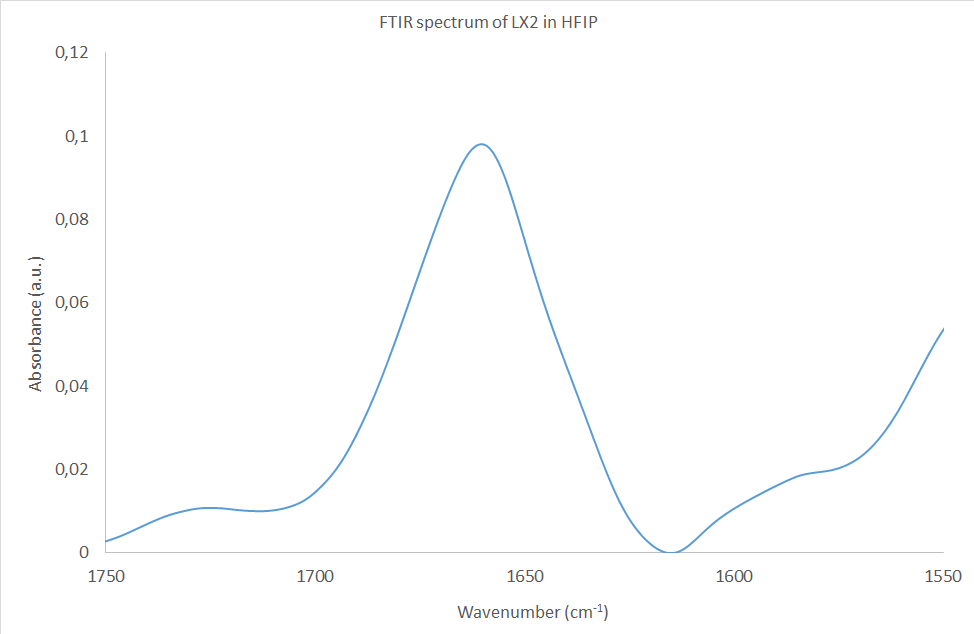

Supplement: S1 Fig — (TIF) [file pone.0166587.s001.tif]

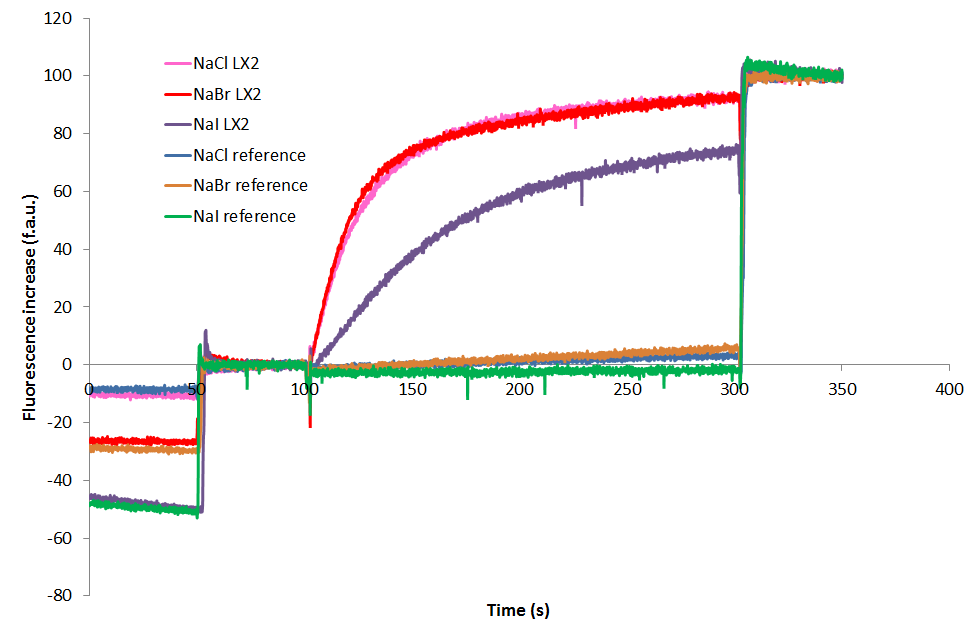

Supplement: S2 Fig — (TIF) [file pone.0166587.s002.tif]

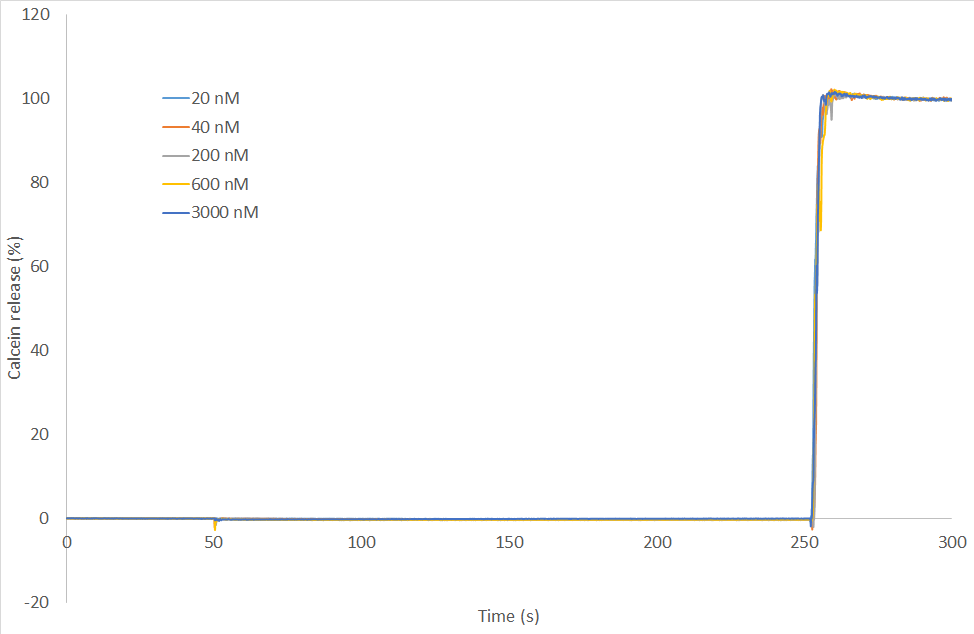

Supplement: S3 Fig — (TIF) [file pone.0166587.s003.tif]
