# Supplementary material for: Membrane Assembly and Ion Transport Ability of a Fluorinated Nanopore
Source: PLoS One. 2016 Nov 11;11(11):e0166587. doi: 10.1371/journal.pone.0166587 (PMC5106009; doi:10.1371/journal.pone.0166587)
Supplement: S1 File — (PDF) [file pone.0166587.s004.pdf]

Parametrization of the trifluoromethylated alanine

Force field parameters for the non-natural trifluoromethylated alanine (ALAF) were built by direct transfer from the CHARMM36 trifluoroethane molecule (TFET) [1] . The bond, angles and dihedral angles involved in the link between trifluoroethane and the peptide backbone were obtained with ParamChem [2,3]. Dihedral angle Cα – Cβ – Cγ – F, corresponding to the rotation of the CF3 group, presented a high parameterization penalty of 50.5. To validate these dihedral angle parameters, a quantum mechanical (QM) energy scan was performed on the shorter molecule trifluoropropane with the ORCA software [4]. Nonbonded parameters were validated by calculating the interaction energy of the trifluoromethylated alanine with a water molecule and comparing the results with those of Chen et al. [1]. The topology and parameters for the trifluoromethylated alanine as used in this work are provided below.

Validation of dihedral angle parameters. Figure S1 compares the results of the trifluoropropane CF3 rotation energy scans obtained with the force field parameters and the quantum mechanical (QM) calculation. The graphics on the Figure S1 (right) show an enlargment of the energy barriers and wells to highlight the close similarity between force field and QM results.

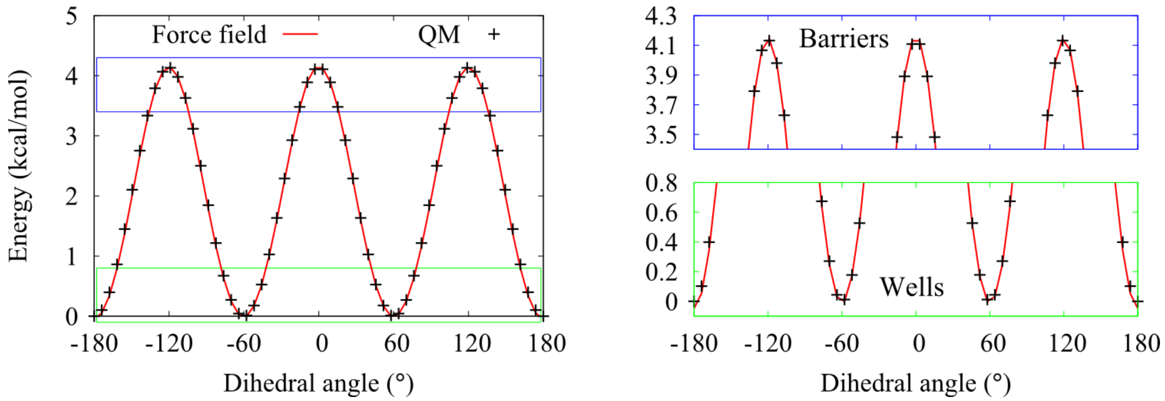

Figure S1: Comparison between the force field and QM energy scans for the rotation of the trifluoropropane CF3 group. On the left, force field and QM results in red curve and black crosses respectively. The blue and green rectangles indicate the regions of energy barriers and wells that are enlarged on the right side of the figure.

Validation of nonbonded parameters. Figure S2 illustrates the two relative orientations of trifluoromethylated alanine and water used to validate nonbonded parameters. Those orientations were chosen following the work of Chen et al. [1]. For the two orientations, the distance between the water and the fluor or carbon was varied to obtain the equilibrium distance

and minimum interaction energy. Since the trifluoromethylated alanine parameters are based on the trifluoroethane, it is expected that the both molecules show a very similar interaction profile.

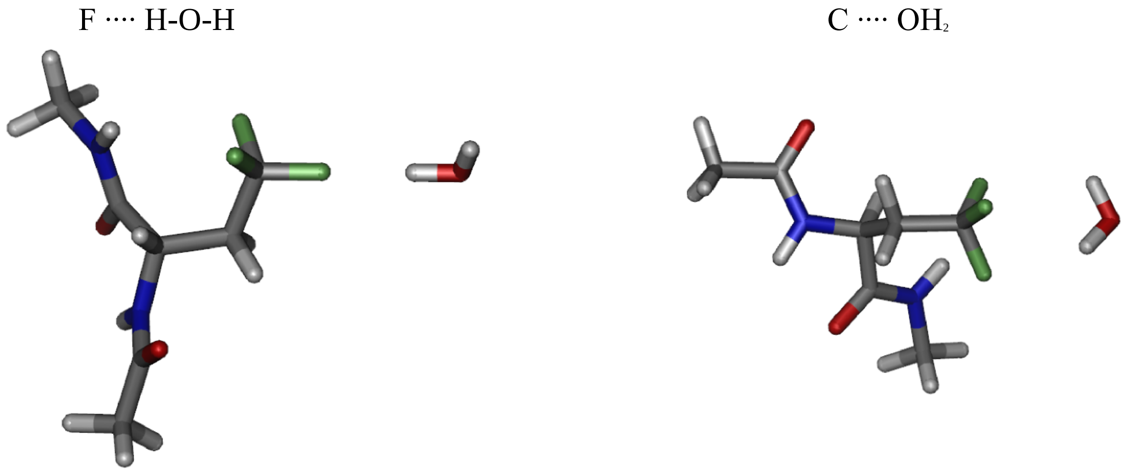

Figure S2: The two orientations used to compute the interaction between trifluoromethylated alanine and water. Fluors are colored green, carbons dark gray, hydrogens light gray, oxygens red and nitrogens blue. On the first orientation (left), one of the water H–O bond is aligned with one of the trifluoromethyl Cγ – F bond, with the hydrogen facing the fluor. One the second orientation (right), the water oxygen is positioned along the Cβ – Cγ bond and the hydrogens are relaxed to their minimum energy position.

Figure S3 shows the force field interactions energy of the trifluoromethylated alanine (ALAF) residue with water as a fonction of distance for both orientations studied. The interaction profile is also compared to that of trifluoroethane (TFET). First, the equilibrium distance and minimum interaction energy obtained for TFET are identical to those given by Chen et al. [1], supporting this validation procedure. Then, the equilibrium distance of ALAF is almost identical to that of TFET for the two studied orientations, as expected. Finally, the minimum interaction energy is 0.2 - 0.3 kcal/mol higher for ALAF. However, this difference is not abnormal and is caused by the slighly more positive partial charge of the amino acid β carbon compared to its equivalent in the trifluoroethane. The nonbonded parameters used in this project are hence valid as they produce exactly the expected water interaction equilibrium distance, and an interaction energy that is very close to that of the trifluoroethane molecule.

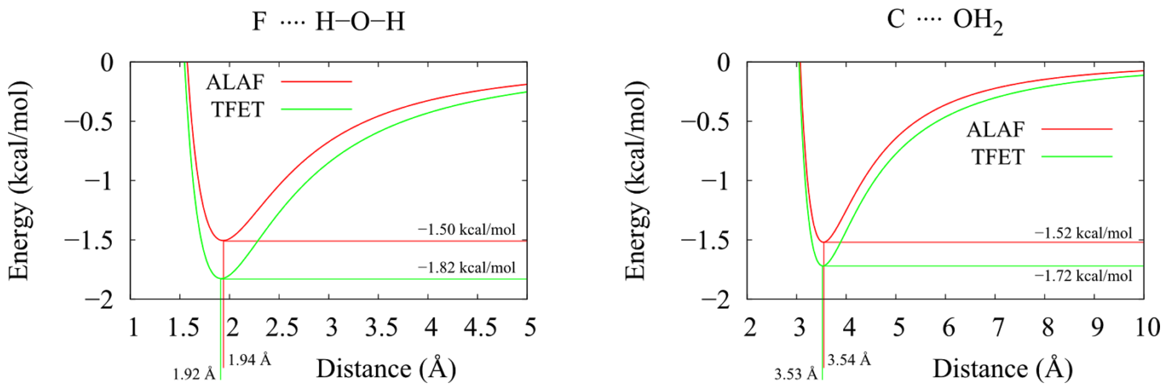

Figure S3: Water interaction energy of ALAF and TFET as a function of distance to water in the two studied orientations. Equilibrium distances and minimum interaction energies are given for each orientation.

REFERENCES

1. Chen, I. J., Daxu, Y., and MacKerell, A.D. Jr. (2002). Combined ab initio/empirical approach for optimization of Lennard-Jones parameters for polar-neutral compounds. J. Comput. Chem. *23*, 199-213.
2. Vanommeslaeghe, K., and MacKerell, A.D. Jr. (2012). Automation of the CHARMM general force field (CGenFF) I: bond perception and atom typing. J. Chem. Inf. Model. *52*, 3144-3154.
3. Vanommeslaeghe, K., Hatcher, E., Acharya, C., Kundu, S., Zhong, S., Shim, J., Darian, E., Guvench, O., Lopes, P., Vorobyov, I., and MacKerell, A.D. Jr. (2010). CHARMM general force field: A force field for drug-like molecules compatible with the CHARMM all-atom additive biological force fields. J. Comput. Chem. *31*, 671-690.
4. Neese, F. (2012). The ORCA program system. Comput. Mol. Sci. *2*, 73-78.
