# Supplementary material for: Membrane Assembly and Ion Transport Ability of a Fluorinated Nanopore
Source: PLoS One. 2016 Nov 11;11(11):e0166587. doi: 10.1371/journal.pone.0166587 (PMC5106009; doi:10.1371/journal.pone.0166587)
Supplement: S2 File — (PDF) [file pone.0166587.s005.pdf]

Topology and parameters of the trifluoromethylated alanine

```
* Parameters generated by analogy by
* CHARMM General Force Field (CGenFF) program version 0.9.7 beta
*

! Penalties lower than 10 indicate the analogy is fair; penalties between 10
! and 50 mean some basic validation is recommended; penalties higher than
! 50 indicate poor analogy and mandate extensive validation/optimization.

BONDS
! C36: CT2  CT1   222.500     1.5380  ,  CGenFF: CG311  CG321   222.50     1.5380
CG321    CT1     222.500     1.5380 ! from C36

ANGLES
!CG302  CG321  CG311    58.35    113.50   11.16   2.56100 ! DR_tfe , from CG311 CG321 CG321, penalty= 12
CG302  CG321  CT1      58.35    113.50   11.16   2.56100 ! CG311 is the CA in original ParamChem molecule (serine-
trifluoromethyl.str)
! C36:  NH3  CT1  CT2    67.700   110.0000
NH3    CT1    CG321    67.700   110.0000 ! from C36
! C36:  HB1  CT1  CT2    35.000   111.0000  ,  CGenFF:   CG321  CG311  HGA1     34.50    110.10   22.53   2.17900
HB1    CT1    CG321    35.000   111.0000 ! from C36
! C36:  CT2  CT1  C      52.000   108.0000  ,  CGenFF:   CG2O1  CG311  CG321    52.00    108.00
CG321  CT1    C        52.000   108.0000 ! from C36
! C36:  HA2  CT2  CT1     26.500    110.10   22.53   2.17900  ,  CGenFF:   CG311  CG321  HGA2     33.43    110.10   22.53
2.17900
CT1    CG321  HGA2     26.500   110.10   22.53   2.17900 ! from C36
! C36:  NH1  CT1  CT2    70.000   113.5000  ,  CGenFF:   CG321  CG311  NG2S1    70.00    113.50
NH1    CT1    CG321    70.000   113.5000 ! from C36
! C36:  CT2  CT1  CC     52.000   108.0000  ,  CGenFF:   CG2O1  CG311  CG321    52.00    108.00
CG321  CT1    CC       52.000   108.0000 ! from C36

DIHEDRALS
!FGA3   CG302  CG321  CG311      0.2500  3     0.00 ! DR_tfe , from FGA3 CG302 CG321 OG311, penalty= 50.5
FGA3   CG302  CG321  CT1        0.2500  3     0.00 ! Optimized by fit on QM energy scan
!CG2O1  CG311  CG321  CG302      0.2000  3     0.00 ! DR_tfe , from CG2O1 CG311 CG321 CG321, penalty= 12
C      CT1    CG321  CG302      0.2000  3     0.00 ! CG2O1 is the C in original ParamChem molecule
!NG2S1  CG311  CG321  CG302      0.2000  3     0.00 ! DR_tfe , from NG2S1 CG311 CG321 CG321, penalty= 12
NH1    CT1    CG321  CG302      0.2000  3     0.00 ! NG2S1 is the N in original ParamChem molecule
!HGA1   CG311  CG321  CG302      0.1950  3     0.00 ! DR_tfe , from HGA1 CG311 CG321 CG321, penalty= 12
HB1    CT1    CG321  CG302      0.1950  3     0.00 ! HGA1 is the HA in original ParamChem molecule
! C36:  CT2  CT1  NH1  C         1.8000  1     0.00  ,  CGENFF:  CG321  CG311  NG2S1  CG2O1      1.8000  1     0.00
C      NH1    CT1    CG321      1.8000  1     0.00 ! from C36
! C36:  NH1  CT1  CT2  HA2       0.2000  3     0.00  ,  CGenFF:  NG2S1  CG311  CG321  HGA2       0.2000  3     0.00
NH1    CT1    CG321  HGA2       0.2000  3     0.00 ! from C36
! C36:  H    NH1  CT1  CT2       0.0000  1     0.00  ,  CGenFF:  CG321  CG311  NG2S1  HGP1       0.0000  1     0.00
H      NH1    CT1    CG321      0.0000  1     0.00 ! from C36
! C36:  HB1  CT1  CT2  HA2       0.2000  3     0.00  ,  CGenFF:  HGA1   CG311  CG321  HGA2       0.1950  3     0.00
HB1    CT1    CG321  HGA2       0.2000  3     0.00 ! from C36
! C36:  O    C    CT1  CT2       1.4000  1     0.00  ,  CGenFF:  OG2D1  CG2O1  CG311  CG321      1.4000  1     0.00
CG321  CT1    C      O          1.4000  1     0.00 ! from C36
! C36:  NH1  C    CT1  CT2       0.0000  1     0.00  ,  CGenFF:  NG2S1  CG2O1  CG311  CG321      0.0000  1     0.00
CG321  CT1    C      NH1        0.0000  1     0.00 ! from C36
! C36:  HA2  CT2  CT1  C         0.2000  3     0.00  ,  CGenFF:  CG2O1  CG311  CG321  HGA2       0.2000  3     0.00
HGA2     CG321    CT1      C    0.2000  3     0.00 ! from C36

IMPROPERS

END
```

```
* Topologies generated by
* CHARMM General Force Field (CGenFF) program version 0.9.7 beta
*
36 1

DECL -CA
DECL -C
DECL -O
DECL +N
DECL +HN
DECL +CA
DEFA FIRS NTER LAST CTER
AUTO ANGLES DIHE


RESI ALAF          0.00  !
GROUP                    !
ATOM N    NH1    -0.47  !     |
ATOM HN   H       0.31  !  HN-N
ATOM CA   CT1     0.07  !     |   HB1    F01
```

```
ATOM HA   HB1      0.09  !     |   |      /
GROUP                    !  HA-CA--CB--C01--F02
ATOM CB   CG321  -0.11  !     |   |      \
ATOM HB1  HGA2    0.09  !     |   HB2    F03
ATOM HB2  HGA2    0.09  !   O=C
ATOM C01  CG302   0.38  !     |
ATOM F01  FGA3   -0.15  !
ATOM F02  FGA3   -0.15  !
ATOM F03  FGA3   -0.15  !
GROUP                    !
ATOM C    C       0.51  !
ATOM O    O      -0.51  !

BOND CB CA  C01 CB  N HN  N  CA
BOND C  CA  C +N  CA HA  CB HB1
BOND CB HB2  C01 F01  C01 F02  C01 F03
DOUBLE   O  C
IMPR N -C CA HN  C CA +N O
CMAP -C  N  CA  C   N  CA  C  +N
IC -C   CA  *N    HN    1.3482 123.5700  180.0000 115.1100  0.9988 ! From LYS in
! top_all36_prot.rtf
IC -C   N    CA   C     1.3482 123.5700  180.0000 107.2900  1.5187
IC N    CA   C    +N    1.4504 107.2900  180.0000 117.2700  1.3478
IC +N   CA  *C    O     1.3478 117.2700  180.0000 120.7900  1.2277
IC CA   C    +N   +CA   1.5187 117.2700  180.0000 124.9100  1.4487
IC N    C   *CA   CB    1.4504 107.2900  122.2300 111.3600  1.5568
IC N    C   *CA   HA    1.4504 107.2900 -116.8800 107.3600  1.0833
IC N    CA   CB   C01   1.4504 111.4700  180.0000 115.7600  1.5435
IC C01  CA  *CB   HB1   1.5435 115.7600  120.9000 107.1100  1.1146
IC C01  CA  *CB   HB2   1.5435 115.7600 -124.4800 108.9900  1.1131
IC CA   CB   C01  F03   1.5568 115.7600  180.0000 113.2800  1.3400
IC F03  CB  *C01  F01   1.5397 113.2800  120.7400 109.1000  1.3400
IC F03  CB  *C01  F02   1.5397 113.2800 -122.3400 108.9900  1.3400

END
```
